# RIG-I aggravates interstitial fibrosis via c-Myc-mediated fibroblast activation in UUO mice

**DOI:** 10.1007/s00109-020-01879-x

**Published:** 2020-02-08

**Authors:** Zhuanli Zhou, Jiayun Ni, Jingyao Li, Chuanbing Huo, Naijun Miao, Fan Yin, Qian Cheng, Dan Xu, Hongyan Xie, Panpan Chen, Peiqing Zheng, Yingying Zhang, Li Zhou, Wei Zhang, Chen Yu, Jun Liu, Limin Lu

**Affiliations:** 1 Department of Physiology and Pathophysiology, School of Basic Medical Sciences, Fudan University, 138 Yixueyuan Road, Shanghai, 200032 China; 2 Department of Nephrology, Tongji Hospital, Tongji University School of Medicine, Shanghai, 200065 China

**Keywords:** Renal fibrosis, Inflammatory cytokines, RIG-I, C-Myc, TGF-β/Smad

## Abstract

**Abstract:**

Progressive tubulointerstitial fibrosis is the common final outcome for all kidney diseases evolving into chronic kidney disease (CKD), whereas molecular mechanisms driving fibrogenesis remain elusive. Retinoic acid-inducible gene-I (RIG-I), an intracellular pattern recognition receptor, is originally identified participating in immune response by recognizing virus RNA. Here, we revealed for the first time that RIG-I was induced in unilateral ureteral obstruction (UUO) and folic acid (FA) renal fibrosis models and moderate-degree renal fibrosis patients. Besides, we found RIG-I was mainly located in renal tubular epithelial cells and promoted the production and release of inflammatory cytokines, such as interleukin (IL)-1β and IL-6 through activation of NF-κB. Inflammatory cytokines released by tubular epithelial cells activated c-Myc-mediated TGF-β/Smad signaling in fibroblasts, which in turn aggravated interstitial fibrosis by promoting fibroblast activation and production of extracellular matrix components (ECM). Deficiency of RIG-I attenuated renal fibrosis by the regulation of inflammatory responses, c-Myc expression, and fibroblast activation. Besides, gene silencing of RIG-I reduced inflammatory cytokines in cultured tubular epithelial cells treated with Angiotensin II. Knockdown of c-Myc or c-Myc inhibitor blocked IL-1β-induced fibroblast activation. Collectively, our study demonstrates that RIG-I plays a significant role in the progress of renal fibrosis via regulating c-Myc-mediated fibroblast activation.

**Key messages:**

• RIG-I was constantly elevated in kidneys from renal fibrotic mice.

• RIG-I facilitated inflammatory cytokine production in tubular epithelial cells.

• RIG-I aggravated renal fibrosis via c-Myc-mediated TGF-β/Smad activation.

## Introduction

Renal tubulointerstitial fibrosis is the end point of almost all progressive chronic kidney disease (CKD) [1]. CKD has become a major public health crisis worldwide, which imposes heavy socioeconomic burdens on both individuals and societies [2, 3]. According to the epidemiological data, the morbidity of CKD in China is about 10.8%. Unfortunately, current therapeutic options for patients with CKD are ineffective to reverse or even inhibit the progress of renal fibrosis. When CKD develops into end-stage renal disease (ESRD), no choice other than dialysis or kidney transplantation can be taken in the clinical setting [4]. In order to provide new therapeutic strategies for CKD patients, it remains urgent to explore the cellular and molecular mechanisms of renal fibrosis. Renal fibrosis is associated with fibroblast activation, phenotypic conversion of tubular epithelial cells, recruitment of inflammatory cells, and excessive production of extracellular matrix components (ECM) [1, 5].

Although inflammation is firstly considered an integral part of the host defense mechanisms in response to the injury, excessive inflammation is an initiating factor of fibrotic diseases [1]. In the injured kidneys, both resident cells and inflammatory cells are the important source of proinflammatory cytokines (such as interleukin (IL)-1β and IL-6). However, the mechanisms of synthesis and release of inflammatory mediators remain poorly defined.

Retinoic acid-inducible gene-I (RIG-I) is a recently identified gene encoding a caspase recruitment domain (CARD)-containing protein which functions as a cytoplasmic RNA sensor responsible for the induction of type I interferon (IFN) [6]. Aside from the antiviral response, recent accumulating evidence has indicated that RIG-I is involved in the regulation of gene expression and cellular functions in non-viral systems such as aging, cell proliferation, apoptosis, and inflammation-related diseases [7–9]. Recent data also demonstrate that RIG-I is involved in various chronic inflammatory diseases [10]. Several lines of evidence indicate that RIG-I is an integral component of a proinflammatory mechanism [11, 12]. According to a previous study, RIG-I functions as a positive regulator for NF-κB signaling [7]. NF-κB is considered to be one ubiquitous transcription factor in innate immunity and inflammatory diseases [13]. NF-κB activation is critical to initiate the release of downstream inflammatory cytokines and growth factors, including IL-1β, IL-6, and tumor necrosis factor-α (TNFα) [14]. However, no attention has been paid on the expression pattern and role of RIG-I in renal inflammation and fibrogenesis.

C-Myc is primarily discovered as a proto-oncogene, which promotes the aggression and dedifferentiated of tumor cells [15, 16]. As a nuclear transcription factor, c-Myc is verified to regulate the expression of more than 15% of human genes and implicates in diverse cellular processes, such as growth, differentiation, death, and angiogenesis [17–19]. A recent report has revealed that IL-1β-dependent signaling drives c-Myc accumulation, which then activates kidney stromal cells and promotes cell proliferation [20]. Our previous observation identified that c-Myc promoted the pathogenesis of renal fibrosis via activation of TGF-β pathway [21]. However, it remains unclear what lies upstream of c-Myc to promote renal fibrosis.

In this study, our data demonstrated that RIG-I was markedly induced in the UUO- and FA-induced fibrotic kidneys and was implicated to NF-κB signaling activation and the synthesis and release of inflammatory cytokines in tubular epithelial cells. The inflammatory cytokines upregulated the expression of c-Myc in fibroblasts, which in turn aggravated renal fibrosis by activating TGF-β/Smad pathway. Knockout of RIG-I notably ameliorated interstitial fibrosis by inhibiting renal inflammation responses, c-Myc elevation, and activation of TGF-β/Smad pathway.

## Materials and methods

### Animal studies

Male C57BL/6J and 129/Sv mice (20–25 g) were purchased from Shanghai SLAC Laboratory Animal Co. Ltd. (Shanghai, China). RIG-I-deficient (RIG-I^−/−^) mice were provided by Shanghai Biomodel Organism Science and Technology Development Co., Ltd. (Shanghai, China) and were on a 129/Sv background. The 8- to 10-week-old male RIG-I^−/−^ or wild-type (WT) 129/Sv mice underwent unilateral ureteral obstruction (UUO) and were killed 3, 7, or 14 days later as described [22]. Mice treated with UUO were randomly divided into three groups: Sham, UUO treated with olive oil, and UUO treated with c-Myc inhibitor 10058-F4 (25 mg/kg body weight; Selleckchem, Houston, Texas) administered via gavage in 0.1 mL/day for 7 days.

### Human renal samples

Renal biopsy samples were obtained from patients undergoing diagnostic evaluation and presenting with moderate fibrosis. All participants provided informed written or verbal consent as appropriate. All patient biopsy samples were approved by the Shanghai Tongji Hospital Ethics Committee K-W-2019-008.

### Western blot

Total tissue or cellular lysate preparation and western blot analysis were performed as described previously [23]. The primary antibodies in the study are as follows. Anti-type I collagen, anti-α-smooth muscle actin (α-SMA), anti-RIG-I, and anti-glyceraldehyde-3-phosphate dehydrogenase (GAPDH) were obtained from Abcam (Cambridge, MA, USA). Anti-IL-6, anti-IL-1β, and anti-c-Myc were obtained from Santa Cruz (Dallas, TX, USA). Anti-c-Myc, anti-phospho-Smad3, anti-Smad3, anti-TGF-β, anti-phospho-p65, and anti-p65 were obtained from Cell Signaling Technology (Beverly, MA, USA). Anti-RIG-I, anti-type I collagen, anti-c-Myc, anti-TGF-β, and anti-fibronectin were obtained from Proteintech (Wuhan, China). Anti-IL-6 was obtained from GeneTex (Irvine, CA, USA). After 4 °C overnight incubation, the membranes were incubated with secondary antibodies from Beyotime. Protein bands were visualized by ECL system (AmerSham Biosciences).

### Quantitative real-time polymerase chain reaction

The procedures were performed as described previously [24]. The PCR primers were synthesized by Sangon Biotech (Shanghai, China). Amplification was performed using the following primers: 5′-GGCATTTCCGTGTTTCTT-3′ (forward) and 5′-GGTGGGCTTGGGATAGTC-3′ (reverse) for mouse RIG-I; 5′-CGGACACACAACGTCTTGGAA-3′ (forward) and 5′-AGGATGTAGGCGGTGGCTTTT-3′ (reverse) for mouse c-Myc; and 5′-AGGTCGGTGTGAACGGATTTG-3′ (forward) and 5′-TGTAGACCATGTAGTTGAGGTCA-3′ (reverse) for mouse GAPDH.

### Histopathologic analyses

Mouse kidneys were collected at 7 days after UUO, fixed in 4% paraformaldehyde (PFA) for 24 h, embedded in paraffin, and sectioned 4-μm thickness. Hemaoxylin-eosin (H&E) and Masson’s trichrome were performed for detecting renal defects and renal fibrosis, respectively, using commercial kits (Beyotime, Shanghai, China) according to the manufacturer’s protocol.

### Immunohistochemical examination

Immunohistochemical staining was performed as described [25]. The primary antibodies including anti-p65 (Cell Signaling Technology), anti-Ki67 (Abcam), anti-RIG-I (Proteintech, Sigma), and anti-type I collagen (Abcam) were used in immunohistochemical analyses.

### Immunofluorescence staining and confocal microscopy

Immunofluorescent staining was performed as described [26]. Double immunostaining for RIG-I (green) and various tubular markers (red) was used to define the location of RIG-I in the kidney. Segment-specific tubular markers were used based on previous studies: proximal tubule, aquaporin-1 (AQP1) (Abcam); distal tubule, calbindin D28k (Santa Cruz); and collecting duct, aquaporin-3 (AQP3) (Santa Cruz). The primary antibody fibronectin (Sigma) was used in this study. Double immunostaining c-Myc (Cell Signaling Technology) and α-SMA (Abcam) was utilized to define the expression of c-Myc in the kidney. Confocal microscopy was performed to examine the nuclear location of p65 (Cell Signaling Technology) in tubular cells treated with Angiotensin II (Ang II).

### Cell culture and treatments

Human proximal tubular epithelial cells (HK-2 cells) and normal rat kidney fibroblasts (NRK-49F cells) were obtained from the Institute of Biochemistry and Cell Biology (Shanghai, China) and cultured as described [27]. Cells were serum starved for 12 h before the experiments. Different stimuli were used in this study: (1) IL-1β (1-10 ng/mL, a final concentration of 5 ng/mL in culture medium, PeproTech EC Ltd., London, UK); (2) Ang II (0.01~1 μM, a final concentration of 0.1 μM in culture medium, ApexBio, Houston, TX); and (3) TGF-β (1~50 ng/mL in culture medium, PeproTech EC Ltd., London, UK).

### RNA interference

Small interference RNAs (siRNAs) were synthesized by Biotend (Shanghai, China). The sense sequence for siRNA-*RIG-I* (human) is 5′-GGGAACGAUUCCAUCACUAdTdT-3′, and for siRNA-*c-Myc* (rat) is 5′-GGAAUCUCGAGUGUAAGGAdTdT-3′. In these experiments, siRNAs were transfected by Lipofectamine RNAiMAX reagent (Thermo Fisher Scientific, 13778030) according to the manufacturer’s protocol. Specific silencing of the targeted gene was confirmed by western blot analysis.

### Cell proliferation assay

NRK-49F cells were plated in 6-well plates. When the cells reached 30~50% confluence, they were serum starved for 12 h and then treated accordingly. EdU assay assessed cell proliferation as previously described [28].

### EdU incorporation

Proliferative cells were pulse labeled for 2 h by intraperitoneal injection of mice with 5-ethynyl-2′-deoxyuridine (EdU, 100 mg/kg). Sections were stained with antibodies against α-SMA (Abcam), followed by EdU staining (BeyoClick EdU Cell Proliferation Kit with Alexa Fluor 594, Beyotime) and Hoechst counterstaining (Hoechst 33342).

### Statistics data

Statistics data are expressed as means ± SE. Student’s *t* test was used to compare between two groups. The significance of the differences in mean values between and within multiple groups was examined by one-way ANOVA plus Tukey’s post-test. *P* < 0.05 was considered statistically significant.

## Results

### RIG-I was upregulated in UUO- and FA-treated kidneys

**Fig. 1:**
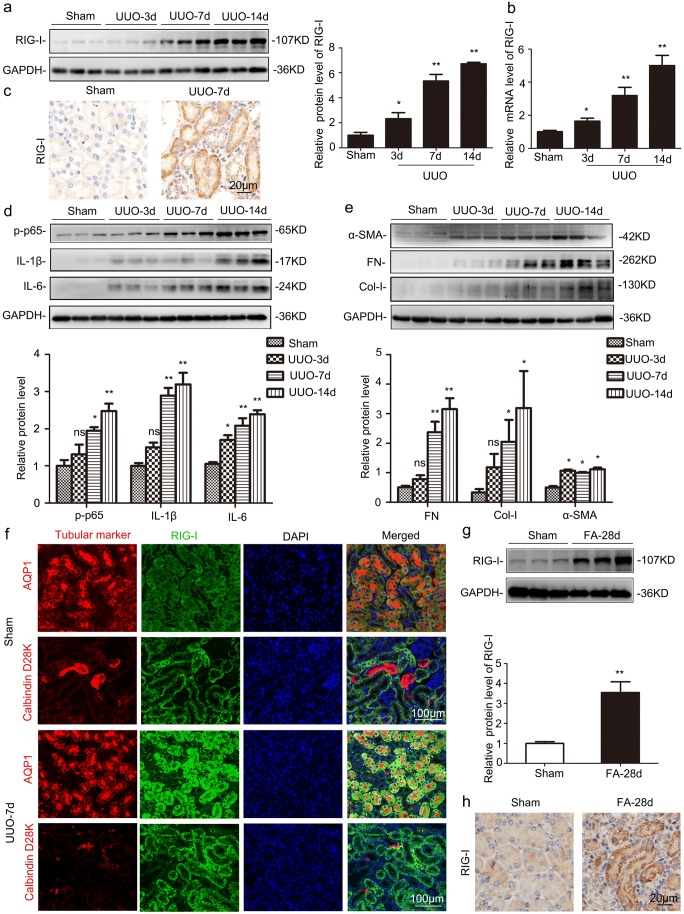
RIG-I was significantly upregulated in UUO- and FA-treated kidneys. **a** Representative western blot analysis of levels of RIG-I in UUO-treated kidneys. *P < 0.05 vs. Sham-operated mice (*n* = 6). **b** Relative mRNA levels of *RIG-I* in UUO-treated kidneys. *P < 0.05 vs. Sham-operated mice (*n* = 4 or 6). **c** Representative images of immunohistochemical staining of RIG-I in UUO-treated kidneys. Original magnification, × 400 (*n* = 4). **d** Western blot was performed to examine the levels of phospho-p65, interleukin (IL)-1β and IL-6. *P < 0.05 vs. Sham-operated mice (*n* = 6). **e** Western blot was performed to examine the protein levels of extracellular matrix components fibronectin (FN), type I collagen (Col-I), and α-smooth muscle actin (α-SMA). *P < 0.05 vs. Sham-operated mice (*n* = 3). **f** Co-immunofluorescence staining for RIG-I and tubular segment-specific markers in UUO-treated kidneys. Markers were used as follows, proximal tubule, aquaporin-1 (AQP1) and distal tubule, calbindin D28k. Original magnification, × 200. **g** Representative western blot of RIG-I in FA-treated kidneys. *P < 0.05 vs. Sham-operated mice (*n* = 3). **h** Representative images of immunohistochemical staining of RIG-I in FA-treated kidneys. Original magnification, × 400 (*n* = 4). GAPDH, glyceraldehyde-3-phosphate dehydrogenase

Classical renal fibrotic animal models induced by UUO were used in this experiment. Western blot, RT-qPCR, and IHC staining analyses showed that RIG-I was significantly induced in UUO-treated kidneys (Fig. 1a–c). Inflammation generally plays a crucial role in the initiation of renal fibrogenesis. Besides, NF-κB cascades are one of the most important signaling pathways priming immunity and inflammation. As shown in Fig. 1d, the phosphorylation level of p65 and the target genes of NF-κB, IL-1β, and IL-6 were all increased in a time-dependent manner in UUO-treated kidneys by western blot analysis. In addition, extracellular matrix components including fibronectin (FN), type I collagen (Col-I), and α-SMA were also time dependently increased in UUO-treated kidneys (Fig. 1e). To validate the tubular segment location of RIG-I expression, we performed double-immunostaining for RIG-I (green) and various tubular markers (red) in the kidney, proximal tubule, aquaporin-1 (AQP1), and distal tubule, calbindin D28k. These data indicated that RIG-I was largely localized in cytoplasm of proximal tubules (Fig. 1f). The upregulation of RIG-I was also detected in FA-treated kidneys (Fig. 1g–h).

### RIG-I deficiency ameliorated UUO-induced interstitial fibrosis

**Fig. 2:**
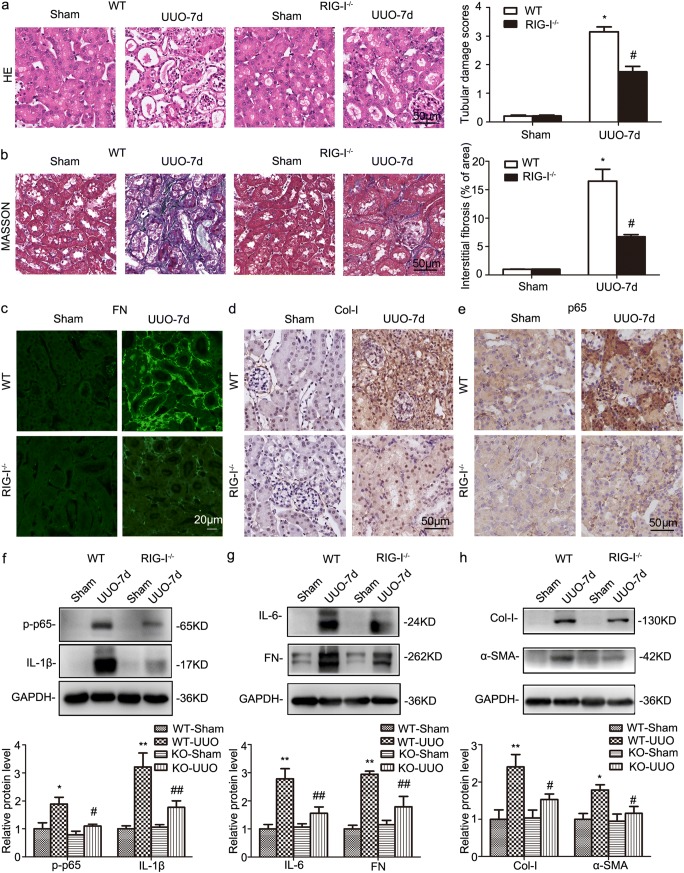
RIG-I deficiency inhibited inflammation response and ameliorated renal interstitial fibrosis in UUO-treated kidneys. **a** Representative micrographs of H&E staining in obstructed kidneys from WT and RIG-I^−/−^ mice. Original magnification, × 400 (*n* = 4). **b** Representative micrographs of Masson’s trichrome staining in obstructed kidneys from WT and RIG-I^−/−^ mice. Original magnification, × 400 (*n* = 4). **c** Representative micrographs of immunofluorescence staining of FN in obstructed kidneys from WT and RIG-I^−/−^mice. Original magnification, × 400 (*n* = 4). **d** Representative micrographs of immunohistochemical staining of Col-I in obstructed kidneys from WT and RIG-I^−/−^mice. Original magnification, × 400 (*n* = 4). **e** Representative images of immunohistochemical staining of p65 in obstructed kidneys from WT and RIG-I^−/−^mice. Original magnification, × 400 (*n* = 4). **f** Representative western blot and quantitative data showing the expression of phospho-p65 and IL-1β in obstructed kidneys from WT and RIG-I^−/−^ mice. *P < 0.05 vs. Sham-operated WT mice; ^#^*P* < 0.05 vs. WT mice with UUO (*n* = 4 or 5). **g** Representative western blot and quantitative data showing the expression of IL-6 and FN in obstructed kidneys from WT and RIG-I^−/−^ mice. *P < 0.05 vs. Sham-operated WT mice; ^#^*P* < 0.05 vs. WT mice with UUO (*n* = 4 or 5). **h** Representative western blot and quantitative data showing the expression Col-I and α-SMA in obstructed kidneys from WT and RIG-I^−/−^ mice. Data are presented as the mean ± SEM (*n* = 5). *P < 0.05 vs. Sham-operated WT mice; ^#^*P* < 0.05 vs. WT mice with UUO. ns, no significant; WT, wild type; KO, knockout

To further identify the role of RIG-I in renal fibrosis, RIG-I^−/−^ mice were deployed and subjected to UUO. As shown in Fig. 2a, WT mice displayed remarkable tubular atrophy 7 days after UUO, whereas much less morphologic injury was observed in RIG-I^−/−^ mice by HE staining analysis. Masson’s trichrome staining revealed that the UUO induced the deposition of extracellular matrix deposition was markedly suppressed by RIG-I abrogation (Fig. 2b). IF indicated that FN had a significant increase in WT mice after UUO, whereas the increase was blunt in RIG-I knockout mice (Fig. 2c). IHC showed that RIG-I^−/−^ mice exhibited a lower level of the deposition of type I collagen than their wild-type (WT) littermates after UUO (Fig. 2d). Besides, IHC staining demonstrated nuclear location of NF-κB was increased in UUO-treated kidneys and eased by RIG-I knockout (Fig. 2e). In contrast with WT mice, knockout of RIG-I dramatically abrogated UUO induced the upregulation of phospho-p65, IL-1β, and IL-6 and extracellular matrix components FN, Col-I, and α-SMA (Fig. 2f–h).

### The c-Myc inhibitor, 10058-F4, attenuated fibrosis significantly in UUO-treated kidneys

**Fig. 3:**
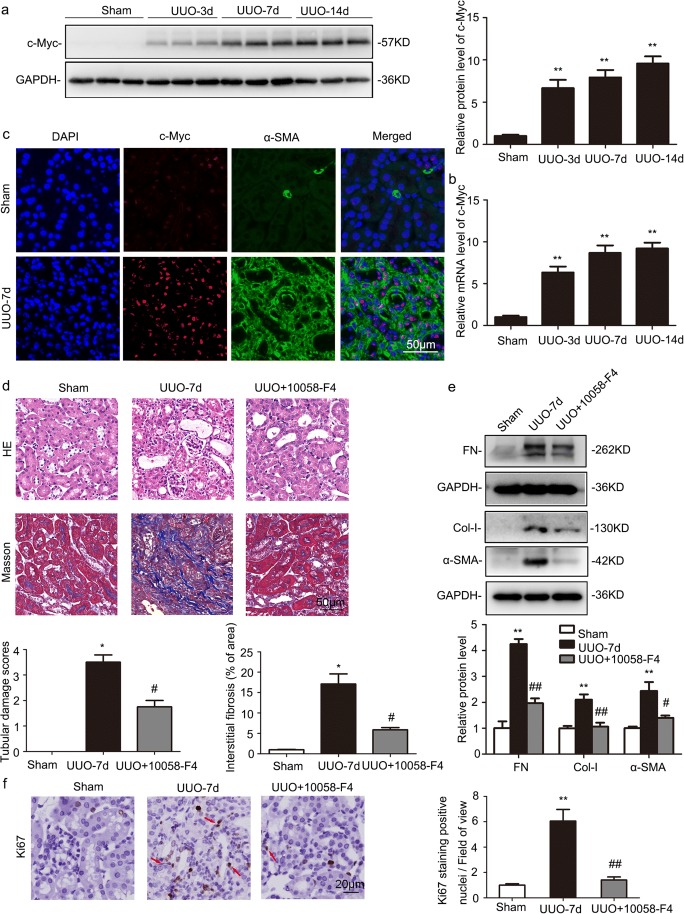
The c-Myc inhibitor, 10058-F4, attenuated renal fibrosis in UUO-treated kidneys. Sham-operated and UUO mice received vehicle or 10058-F4 (25 mg/kg body weight), respectively, via oral gavage at 0.1 mL/day for 7 days (*n* = 6). **a** Representative western blot analysis of renal protein level of c-Myc normalized to GAPDH. *P < 0.05 vs. Sham-operated mice (*n* = 6). **b** Relative mRNA levels of *c-Myc* in UUO-treated kidneys. *P < 0.05 vs. Sham-operated mice (*n* = 4). **c** Co-immunofluorescence staining for c-Myc (red) and α-SMA (green) in UUO-treated kidneys. Original magnification, × 400 (*n* = 4). **d** Representative hematoxylin-eosin (H&E) and Masson’s trichrome-stained kidneys. Original magnification, × 400 (*n* = 4). **e** Representative western blot analysis of renal protein level of FN, Col-I, and α-SMA normalized to GAPDH. *P < 0.05 vs. Sham-operated mice. ^#^*P* < 0.05 vs. UUO with vehicle (*n* = 3). **f** Immunostaining for positive Ki67 nuclei in kidney sections. Original magnification, × 400. Quantification of Ki67-positive nuclei per field of view in (**e**). *P < 0.05 vs. Sham-operated mice. ^#^*P* < 0.05 vs. UUO with vehicle (*n* = 5 or 6)

Consistent with previous study, c-Myc was time dependently increased in UUO-treated kidneys after 3 days by western blot and RT-qPCR analyses (Fig. 3a, b). Double immunostaining for c-Myc (red) and α-SMA (green) showed that the expression of c-Myc was enhanced in UUO-treated kidneys and nearly completely colocalized with α-SMA, a marker of fibroblasts (Fig. 3c).

Compared with UUO mice under 10058-F4 treatment, UUO mice only treated with vehicle showed more severe renal tubular injury. severe renal tubular injury, as evidenced by the loss of brush border, tubule dilatation, and tubular cast formation by HE staining analysis and more extracellular matrix deposition by Masson’s trichrome staining analysis (Fig. 3d). Western blot revealed that UUO-induced increase in FN, Col-I, and α-SMA were effectively suppressed by 10058-F4 treatment (Fig. 3e). Furthermore, the UUO-induced fibroblast proliferation was lessened following 10058-F4 treatment by Ki67 immunostaining analysis (Fig. 3f).

### RIG-I deficiency downregulated the expression of c-Myc and fibroblast proliferation in UUO-treated kidneys

**Fig. 4:**
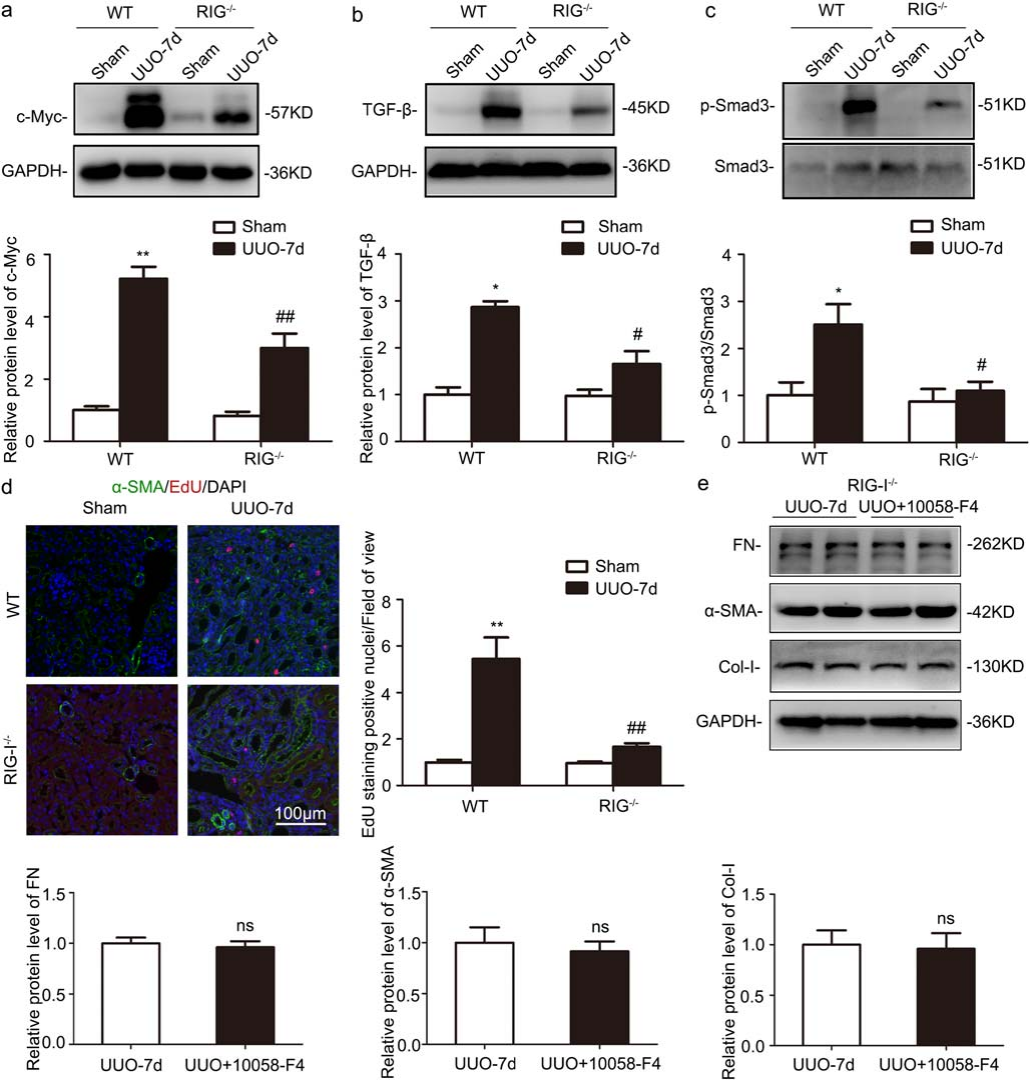
RIG-I deficiency attenuated renal fibrosis by inhibiting c-Myc-mediated TGF-β/Smad signaling. **a** Representative western blot and quantitative data showing the expression of c-Myc in UUO-treated kidneys from WT and RIG-I^−/−^ mice. *P < 0.05 vs. Sham-operated WT mice; ^#^*P* < 0.05 vs. WT mice with UUO (*n* = 5). **b** Representative western blot and quantitative data showing the expression of TGF-β in UUO-treated kidneys from WT and RIG-I^−/−^ mice. *P < 0.05 vs. Sham-operated WT mice; ^#^*P* < 0.05 vs WT mice with UUO (*n* = 3). **c** Representative western blot and quantitative data showing the level of phosphorylated (*p*)-Smad3/Smad3 in UUO-treated kidneys from WT and RIG-I^−/−^ mice. *P < 0.05 vs. Sham-operated WT mice; ^#^*P* < 0.05 vs. WT mice with UUO (*n* = 3). **d** IF images and quantitative data for EdU in UUO-treated kidneys from WT and RIG-I^−/−^ mice. Arrows indicate fibroblasts that are positive for EdU. Original magnification, × 200. *P < 0.05 vs. Sham-operated WT mice; ^#^*P* < 0.05 vs. WT mice with UUO (*n* = 6). **e** Representative western blot and quantitative data showing the expression of FN, Col-I, and α-SMA in UUO-treated kidneys from RIG-I^−/−^ mice with or without 10058-F4. *P < 0.05 vs. Sham-operated WT mice; ^#^*P* < 0.05 vs. WT mice with UUO (*n* = 5)

Western blot analysis of kidney lysates revealed that c-Myc was significantly upregulated following UUO, whereas the increase was blunt in RIG-I^−/−^ mice (Fig. 4a). TGF-β/Smad pathway is essential in mediating the profibrotic effect of c-Myc. The levels of TGF-β and the phosphorylated (p)-Smad3 were markedly increased in the UUO kidneys, whereas RIG-I^−/−^ deficiency suppressed the upregulation compared those from WT mice (Fig. 4b, c). EdU incorporation assay demonstrated RIG-I depletion effectively inhibited the UUO-induced fibroblast proliferation (Fig. 4d). Furthermore, WB revealed 10058-F4 had no additional effects on the expression of extracellular matrix components including FN, Col-I, and α-SMA after abrogation of RIG-I (Fig. 4e). These results further revealed that RIG-I deficiency alleviated renal fibrosis, at least in part, by c-Myc-mediating TGF-β/Smad signaling.

### Gene silencing of RIG-I suppressed the expression of proinflammatory cytokines in HK-2 cells after angiotensin II treatment

**Fig. 5:**
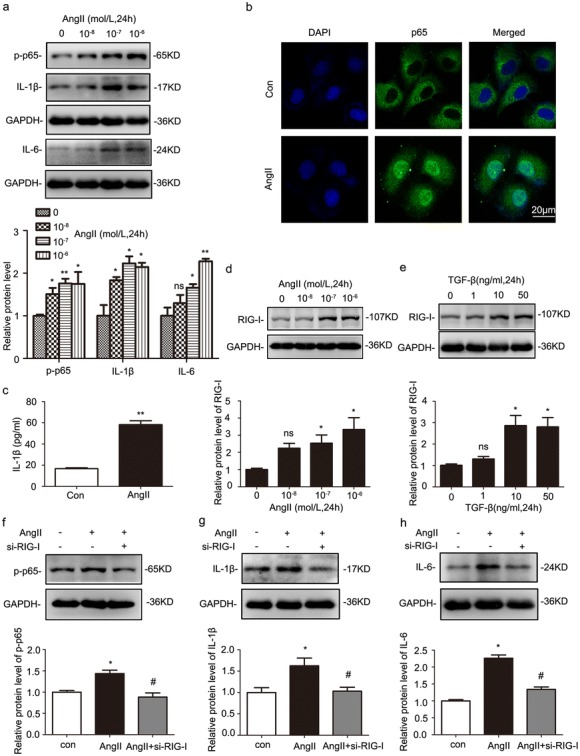
Gene silencing of RIG-I suppressed the release of proinflammatory cytokines in Ang II-treated human renal proximal tubule cells (HK-2 cells). **a** Western blot was performed to examine the expression of phospho-p65, IL-1β, and IL-6 in HK-2 cells with Ang II treatment. *P < 0.05 vs. 0 mol/L (*n* = 4). **b** The nuclear location of p65 in HK-2 cells after Ang II treatment was visualized using confocal microscopy. Original magnification, × 400 (*n* = 4). **c** ELISA was used to detect the release of IL-1β in HK-2 cells after Ang II treatment. *P < 0.05 vs. Con (*n* = 4). **d** Representative western blot analyses of the expression of RIG-I in Ang II-stimulated HK-2 cells. *P < 0.05 vs. 0 mol/L (*n* = 3). **e** Representative western blot analyses of the expression of RIG-I in transforming growth factor-β (TGF-β)-stimulated HK-2 cells. *P < 0.05 vs. 0 ng/mL (*n* = 3). **f**–**h** Western blot analyses of the effects of gene silencing of RIG-I on the levels of phospho-p65, IL-1β, and IL-6 in HK-2 cells under Ang II treatment. *P < 0.05 vs. control (*n* = 3); ^#^*P* < 0.05 vs. NC (under Ang II treatment); Con, control

It is well documented that renin-angiotensin system (RAS) plays a vital role in processive renal fibrogenesis after injury. Ang II is the most important effectors of RAS. Therefore, we treated HK-2 cells with different Ang II concentrations. Western blot results demonstrated the expression of phospho-p65, IL-1β, and IL-6 was increased in a dose-dependent manner under Ang II treatment (Fig. 5a). Immunocytochemistry (ICC) results showed that nuclear p65 staining was remarkably enhanced in tubular cells after Ang II treatment (Fig. 5b). In addition, ELISA revealed that the release of IL-1β was increased after Ang II treatment (Fig. 5c). These results suggest that RAS promotes NF-κB signaling activation and inflammatory cytokine release from tubular epithelia cells.

To identify the role of RIG-I in inflammatory activation, HK-2 cells were starved in serum-free medium for 12 h and then treated with different doses of Ang II and TGF-β for 24 h. The level of RIG-I was upregulated in a dose-dependent manner (Fig. 5d, e). Besides, the expression of phospho-p65, IL-1β, and IL-6 were elevated with Ang II treatment for 24 h. Silencing RIG-I gene via small interfering RNA effectively blunt Ang II effects, which inhibited the upregulation of phospho-p65, IL-1β, and IL-6 (Fig. 5f–h).

### Gene silencing of c-Myc diminished IL-1β-induced fibroblast proliferation and activation through inhibiting TGF-β/Smad signaling

**Fig. 6:**
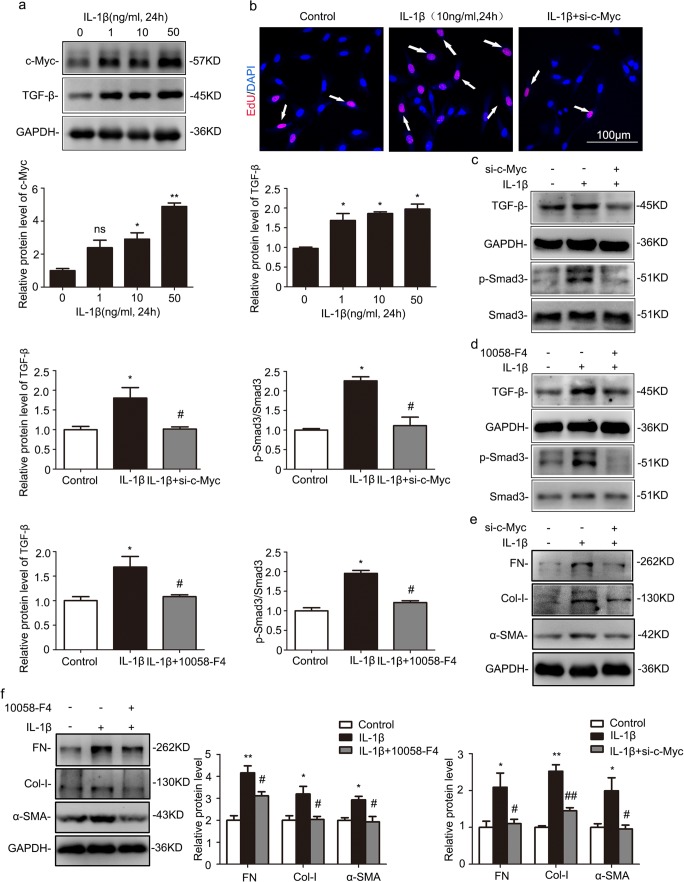
Gene silencing of c-Myc abolished IL-1β-induced profibrotic responses by inhibiting TGF-β/Smad signaling in NRK-49F cells. Cells were treated with IL-1β in the presence or absence of *c-Myc* small interfering RNA (siRNA) or c-Myc inhibitor, 10058-F4. **a** Representative western blot and quantitative data showing increased protein levels of c-Myc and TGF-β in NRK-49F cells with different IL-1β dose treatment for 24 h. *P < 0.05 vs. 0 ng/mL (*n* = 3 or 6). **b** EdU assay showing the effects of gene silencing of c-Myc on fibroblast proliferation. Original magnification, × 200 (*n* = 4). **c** Representative western blot and quantitative data showing the effects of gene silencing of c-Myc on the levels of TGF-β, p-Smad3, and Smad3 in NRK-49F cells with IL-1β treatment. *P < 0.05 vs. control; ^#^*P* < 0.05 vs. NC (under IL-1β treatment) (*n* = 3). **d** Representative western blot and quantitative data showing the effects of 10058-F4 on the levels of TGF-β, p-Smad3, and Smad3 in NRK-49F cells with IL-1β treatment. *P < 0.05 vs. control; ^#^*P* < 0.05 vs. NC (under IL-1β treatment) (*n* = 3). **e** Representative western blot and quantitative data showing the effects of gene silencing of c-Myc on the levels of FN, Col-I, and α-SMA in NRK-49F cells with IL-1β treatment. *P < 0.05 vs. control; ^#^*P* < 0.05 vs. NC (under IL-1β treatment) (*n* = 3 or 6). **f** Representative western blot and quantitative data showing the effects of 10058-F4 on the levels of FN, Col-I, and α-SMA in NRK-49F cells with IL-1β treatment. *P < 0.05 vs. control; ^#^*P* < 0.05 vs. NC (under IL-1β treatment) (*n* = 3). NC, negative control; EdU, 5-ethynyl-2′-deoxyuridine

It is well accepted that the activation of interstitial fibroblasts plays an essential role in renal fibrogenesis. In cultured normal rat kidney fibroblast (NRK-49F) cells, the protein levels of c-Myc and TGF-β were enhanced in a dose-dependent manner under IL-1β treatment (Fig. 6a). EdU assay revealed that knockdown of c-Myc inhibited IL-1β-induced fibroblast proliferation (Fig. 6b). To elucidate the mechanism of c-Myc in kidney fibrosis, we also examined the downstream of c-Myc, TGF-β pathway. Western blot demonstrated treating NRK-49F cells with IL-1β for 24 h increased in the levels of TGF-β and phospho-Smad3/total Smad3, but this upregulation was blocked by either gene silencing of c-Myc or the c-Myc inhibitor, 10058-F4 (Fig. 6c, d). Moreover, either c-Myc knockdown or 10058-F4 dramatically abrogated the upregulation of FN, Col-I, and α-SMA in IL-1β-treated NRK-49F cells (Fig. 6e, f). These data indicated the inflammatory cytokines activated fibroblasts via c-Myc-mediated TGF-β/Smad pathway.

### RIG-I was increased in sections of kidney biopsy samples from patients with moderate fibrosis

**Fig. 7:**
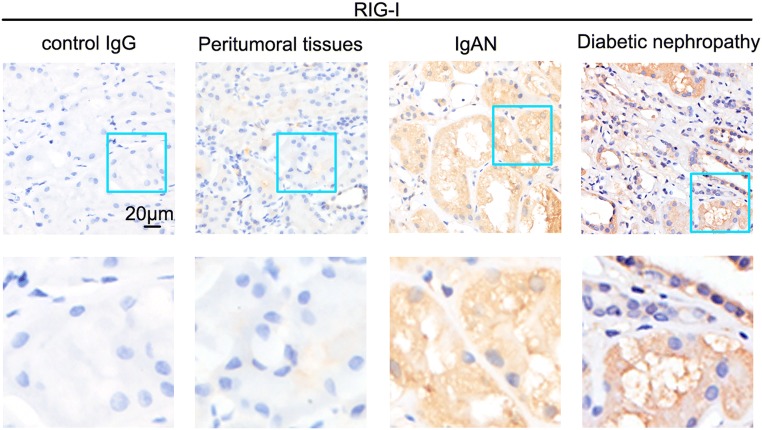
RIG-I was upregulated in moderate-degree fibrosis patients. Representative images of immunohistochemical staining of RIG-I in the kidney from patients with diabetic nephropathy or IgA nephropathy

As shown in Fig. 7, we further confirmed the increase of RIG-I in kidney from patients presenting with moderate fibrosis by IHC staining analyses, which was in accordance with animal experimental models.

## Discussion

Renal tubulointerstitial fibrosis is considered, by and large, to be a failed wound-healing process and a crucial determinant leading to ESRD [29]. However, the underlying mechanism of fibrogenesis warrants further investigation. Acquiring better therapies in patients relies on better understanding of the molecular mechanism modulating fibrogenic events. RIG-I is firstly identified as a member of RIG-I-like receptors (RLRs) for recognizing cytoplasmic viral RNA and resulting immunological responses [30, 31]. An increasing number of studies have shown that RIG-I also plays an important role in cell proliferation, apoptosis, and inflammatory diseases [32, 33]. It is reported that RIG-I participates in the pathogenesis of different types of cancer including acute myeloid leukemia, nasopharyngeal carcinoma, and hepatocellular carcinoma [9, 34, 35]. The intracellular klotho inhibits RIG-I-induced expression of IL-6 and IL-8 by directly interacting with RIG-I [10]. Besides, it is indicated that RIG-I functions as a positive regulator for NF-κB signaling [7]. Previous studies reveal that activation of NF-κB could directly facilitate fibroblast activation and renal fibrosis [36]. Thus, we speculated that RIG-I may be involved in fibrogenesis by implicating NF-κB signaling activation.

We found that RIG-I expression was barely detectable in normal kidneys but was markedly upregulated in renal tubules following UUO administration. Besides, the levels of phospho-p65, IL-1β, and IL-6 were induced in the UUO-treated kidneys. Double immunostaining of RIG-I and tubular markers demonstrated that RIG-I was mainly located in proximal tubules. Moreover, the upregulation of RIG-I was also observed in FA-treated mice. These data suggested that RIG-I was associated with renal fibrosis. To further explore the role of RIG-I in renal fibrosis, we deployed RIG^−/−^ mice subjected to UUO. Compared with WT mice, RIG-deficient mice showed histologically moderate renal injuries during UUO. At the molecular levels, the levels of phospho-65, IL-1β, IL-6, and ECM proteins (FN, Col-I, and α-SMA) were much lower than in WT UUO mice. Besides, both the phosphorylation level and the nuclear translocation of p65 were abrogated in RIG-I-deficient mice. Our results demonstrated that RIG-I facilitated renal fibrosis via activating NF-κB signaling mediating inflammatory responses in UUO mice.

It is widely known that c-Myc, as a multifunctional transcription factor, controls wide-ranging biologic processes such as cell proliferation, transformation, and apoptosis [37, 38]. Our previous studies showed that c-Myc promoted renal fibrosis by stimulating TGF-β pathway [21]. However, it remains unclear what lies upstream of c-Myc to promote renal fibrosis. In this study, we demonstrated that deficiency of RIG-I suppressed the expression of c-Myc in UUO mice. TGF-β/Smad pathway played a main role in c-Myc mediating fibrotic process. In the RIG**-**I^−/−^ mice, our data also showed the increase of TGF-β and p-Smad3/Smad3 was suppressed significantly in RIG-I-deficient UUO mice. However, the c-Myc inhibitor, 10058-F4, had no additional beneficial effects on fibrosis after knockout of RIG-I. Thus, we considered that RIG-I exacerbated renal fibrosis via c-Myc and its downstream TGF-β/Smad pathway.

Intrarenal RAS activation plays a key role in renal fibrogenesis [39]. Ang II is regarded as a key effective mediator in RAS-induced renal fibrosis. Much evidence indicated Ang II induced pro-fibrotic and pro-inflammatory responses in renal tubular epithelial cells [39]. However, the mechanism of Ang II triggering inflammation was poorly understood. We found that in cultured HK-2 cells, the expression of p-p65, IL-1β, and IL-6 was remarkably induced in a dose-dependent manner following Ang II treatment. Furthermore, the release of IL-1β and the nuclear location of p65 were also increased in HK-2 cells after Ang II treatment. In this study, we found that RIG-I was induced in HK-2 cells after Ang II and TGF-β administration. In cultured HK-2 cells, knockdown on RIG-I remarkably inhibited the expression of phospho-p65, IL-1β, and IL-6 induced by Ang II, indicating that RIG-I was associated with Ang II-induced inflammatory cascade activation.

IL-1β, a major innate inflammatory cytokine, is able to trigger cell stress and the synthesis of other inflammatory cytokines [40]. According to previous studies, inflammatory cytokine IL-1β signaling has been implicated in stabilization of Myc and upregulation of Myc target genes [20, 41]. The association between inflammation and fibrosis has been established in previous investigations [42]. However, the cellular and molecular mechanisms between inflammatory signals and fibrogenesis remain to be further explored. Fibroblasts participated in the priming and progression of renal fibrosis by producing excessive fibrotic matrix [42]. We believe that resident fibroblasts acquire a myofibroblast phenotype by profibrotic cytokines secreting tubular cells after being damaged. Therefore, we stimulated NRK-49F cells with IL-1β in vitro. As we expected, the expression of c-Myc and TGF-β in NRK-49F cells was increased in a dose-dependent manner under IL-1β treatment. Gene silencing of c-Myc effectively reversed the effects of IL-1β on NRK-49F cells, including fibroblast proliferation, activation of TGF-β/Smad signaling, and the production of ECM proteins. Consequently, we believed that inflammatory cytokines like IL-1β released by tubular epithelial cells induced the expression of c-Myc and TGF-β/Smad signaling in fibroblasts.

In the study, we demonstrated the potential role of RIG-I in renal fibrosis through the activation of NF-κB signaling in tubular epithelial cells. RIG-I is initially identified as a cytoplasmic receptor for recognizing viral dsRNA. Here, our data showed RIG-I could also be induced in non-viral infection condition. However, the exact mechanism for upregulation of RIG-I other than virus infection needed to be further explored.

**Fig. 8:**
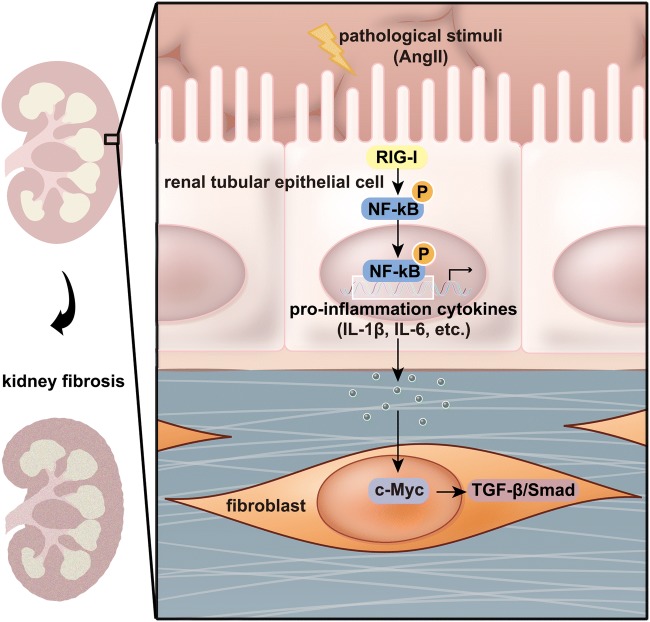
The mechanisms of RIG-I promoted renal interstitial fibrosis. The expression of RIG-I was induced under pathological stimulation, which activated NF-κB signaling and promoted the synthesis and release of proinflammatory mediators in renal tubular epithelial cells. The proinflammatory cytokines released by renal tubular epithelial cells dramatically enhanced the expression of c-Myc in fibroblasts, which activated TGF-β/Smad signaling-mediated the proliferation and activation of fibroblasts

In conclusion, this study for the first time investigates the role of RIG-I in renal fibrosis. We confirm that RIG-I promotes fibrogenesis by the activation of NF-κB and synthesis and release of proinflammatory cytokines in tubular epithelium cells. The proinflammatory cytokines synthesized and released by tubular epithelium cells activate c-Myc-mediated TGF-β/Smad signaling in fibroblasts, which aggravates renal fibrosis through promoting fibroblast proliferation and overproduction of fibrotic matrix (Fig. 8). It suggests that inhibition of inflammation responses originally induced by RIG-I might be a potential target for the treatment of patients with CKD.
